# Fighting COVID-19 in China’s Greater Bay Area: how do different policy styles affect policy effectiveness?

**DOI:** 10.3389/fpubh.2025.1654076

**Published:** 2025-09-23

**Authors:** Zhuojun Liu, Yongquan Qiu, Yingying Ma

**Affiliations:** ^1^School of Public Administration, South China Agricultural University, Guangzhou, China; ^2^Guangdong Urban and Rural Societal Risk and Emergency Governance Research Center, Guangzhou, China

**Keywords:** policy effectiveness, policy style, public sentiment, COVID-19, Greater Bay Area, China

## Abstract

**Background:**

The COVID-19 pandemic has highlighted how political systems influence public health policy effectiveness. This study examines how different governance styles within China’s Greater Bay Area shaped pandemic responses, comparing Guangdong Province, Hong Kong Special Administrative Region (SAR), and Macau SAR as representatives of society-mobilizing, market-facilitated, and targeted-control styles, respectively. This quasi-natural experiment setting controls for cultural and geographic variables while allowing for meaningful institutional comparison.

**Methods:**

Using a mixed-methods approach, the study analyzed COVID-19 infection statistics and 75,870 social media posts from Weibo and X (December 2019–December 2022). The analysis employed statistical methods, sentiment analysis via the Baidu Application Programming Interface (API), and Latent Dirichlet Allocation (LDA) topic modeling to examine policy styles, effects, and public feedback. Interrupted time series (ITS) analysis was also applied to assess policy impacts across three distinct pandemic phases.

**Results:**

Guangdong’s society-mobilizing approach maintained stable case numbers (mean = 31.03 daily cases in Guangzhou) with the highest sentiment scores (0.54) and 51.64% positive reactions. Hong Kong’s market-facilitated approach showed the highest infection rates (mean = 442.95) and lowest sentiment scores (0.46). Macau’s targeted-control approach achieved the lowest infection rates (mean = 0.79) with moderate sentiment scores (0.47). Interrupted time series (ITS) analysis revealed distinct transmission trends in each region, with significant changes observed during the Omicron phase in Guangzhou and Hong Kong, and sustained low transmission in Macau. Topic modeling identified region-specific concerns: overseas case imports (Guangdong), vaccine and local case monitoring (Hong Kong), and casino impacts (Macau).

**Conclusion:**

The study demonstrates that effective pandemic response depends on governance–society alignment, particularly during early outbreaks. While all three systems achieved relative success, their effectiveness varied based on institutional capacity, suggesting that successful crisis management requires consideration of political social structures while maintaining adaptability in transitioning from containment to endemic management. These findings offer a transferable framework for evaluating governance effectiveness in public health crises beyond the Greater Bay Area.

## 1 Introduction

The COVID-19 pandemic has thrust the political foundations of public health policy into the spotlight, revealing the pivotal role that political systems play in crafting and executing effective disease control strategies. It is evident that political structures significantly shape the success of public health initiatives, particularly in times of crisis (1). The diverse political landscapes across the globe have led to a wide range of responses to COVID-19, underscoring the urgent need to analyze these differences. Understanding these variations is crucial for enhancing our preparedness and response to potential future pandemics (2–4). It is of paramount importance to navigate the complex interplay between governance and public health.

According to previous studies, COVID-19 anti-epidemic policies have demonstrated significant differences between authoritarian and democratic systems, shaped by governance structures and societal values. It is argued that authoritarian regimes like China implemented stringent measures rapidly, exemplified by the “zero-COVID (qingling)” policy, to reinforce regime legitimacy, often at the expense of public trust and prioritizing state control over individual freedoms (5). Although these swift actions sometimes result in lower infection and death rates (6, 7), their effectiveness is complicated by transparency issues, as such governments may underreport cases and fatalities (8). This underreporting of infections is a widespread global challenge, with studies showing that in many countries only a small fraction of symptomatic cases are officially recorded, severely hindering accurate assessment and response efforts (9). On the other side, democracies faced constraints from legal frameworks and public opinion, which initially led to less stringent measures but evolved into more aggressive health policies as the pandemic progressed (10, 11). Some scholars suggest that compliance with public health measures was often higher in democracies, driven by a sense of accountability and solidarity among citizens (7). The varied responses in democratic nations, such as the United States and South Korea, highlight the influence of leadership styles and political cohesion on crisis management (12). While authoritarian regimes may appear more decisive, their responses are often obscured by a lack of transparency, whereas democracies depend on public trust and cooperation to navigate crises effectively (8, 13). It was also argued that China’s political system reflects a unique combination of collective values and governance principles that transcend simple authoritarian categorization, while collective cultural values have contributed to generating a distinctive form of political organization that garners broad societal support (14, 15). Although the existing literature has acknowledged the influence of political institutions on pandemic responses, it still lacks comprehensive cross-regional comparative studies, indicating a pressing need for further analysis of the effectiveness of anti-epidemic policies in countries prioritizing unified social action like China.

This study addresses the research gap by examining how different political systems influenced COVID-19 responses, providing a quasi-natural experiment setting where cultural and geographical factors remain relatively constant in China’s Greater Bay Area. Focusing on Guangdong, Hong Kong SAR, and Macau SAR, we analyze how their distinct governance styles—society-mobilizing, market-facilitated, and targeted-control approaches, respectively—shaped their pandemic policies and outcomes through a “policy style-effect-feedback” framework. The concepts of policy style, policy effectiveness, and policy feedback are interrelated elements within the field of public policy analysis. Policy style refers to a government’s approach to problem-solving and its interactions with various actors in the policymaking process, characterized by features such as bureaucratic accommodation and negotiation, which can complicate policy change (16). Policy effectiveness pertains to how well a policy achieves its intended outcomes, influenced by design choices and the political context in which it operates, particularly measured here through epidemiological indicators such as infection rates and transmission trends (17). Policy feedback describes the dynamic process through which enacted policies reshape political landscapes, public opinion, and institutional behaviors, leading to either self-reinforcing or self-undermining effects that can facilitate or hinder future reforms (18, 19). Together, these concepts underscore the complexity of policymaking and the importance of understanding the long-term implications of policy decisions.

Drawing on comparative crisis-governance studies (20, 21), we view political systems as antecedents that shape institutional capacity by influencing the speed, scale, and legitimacy with which each style can be deployed, thus determining both the tools available and the public’s willingness to comply. The three governance styles reflect clear theoretical differences. The society-mobilizing approach activates broad public participation through strong state leadership and collective action (22, 23). The market-facilitated approach relies on decentralized coordination, using market mechanisms and private sector roles to support pandemic response (24, 25). The targeted-control approach applies precise, data-driven interventions like localized testing and containment to efficiently manage outbreaks with minimal disruption (26, 27). These approaches build upon the institutional infrastructure theory proposed by previous studies, which emphasizes how long-term institutional foundations shape crisis responses in East Asia (28). The “policy style-effect-feedback” framework captures how policy styles influence infection outcomes and public sentiment while demonstrating how these outcomes inform policy adjustments. Our analysis reveals distinct trajectories, with Hong Kong experiencing the highest cumulative cases, Guangzhou showing moderate containment, and Macau maintaining the lowest numbers. This paper also utilizes official API interfaces provided by the platforms and sentiment analysis to process social media data from Weibo and X (formerly known as Twitter), revealing how public response aligned with each region’s governance approach—stable positive sentiment in Guangdong, predominantly negative responses in Hong Kong, and mixed reactions in Macau with moderate sentiment levels. Building on these findings, we focus on the “policy style-effect-feedback” framework throughout the paper to explain governance approaches and their effectiveness in managing public health crises. To address how political systems mechanistically shape policy outcomes, we also highlight three key factors as supplements: institutional capacity, policy tools, and public response. By examining these distinct approaches within the same geographical area, we contribute to a broader understanding of how governance structures influence crisis management, especially in complex institutional environments where traditional democratic-authoritarian categories do not fully capture the nuances of policy implementation and effectiveness. Political systems shape institutional capacity, which determines how quickly and legitimately resources can be mobilized. This capacity influences the tools available to each policy style and affects public response, which ultimately shapes epidemiological outcomes. These three factors complement the policy style-effect-feedback loop we apply throughout the paper.

## 1 Materials and methods

### 1.1 Study area

**Figure 1:**
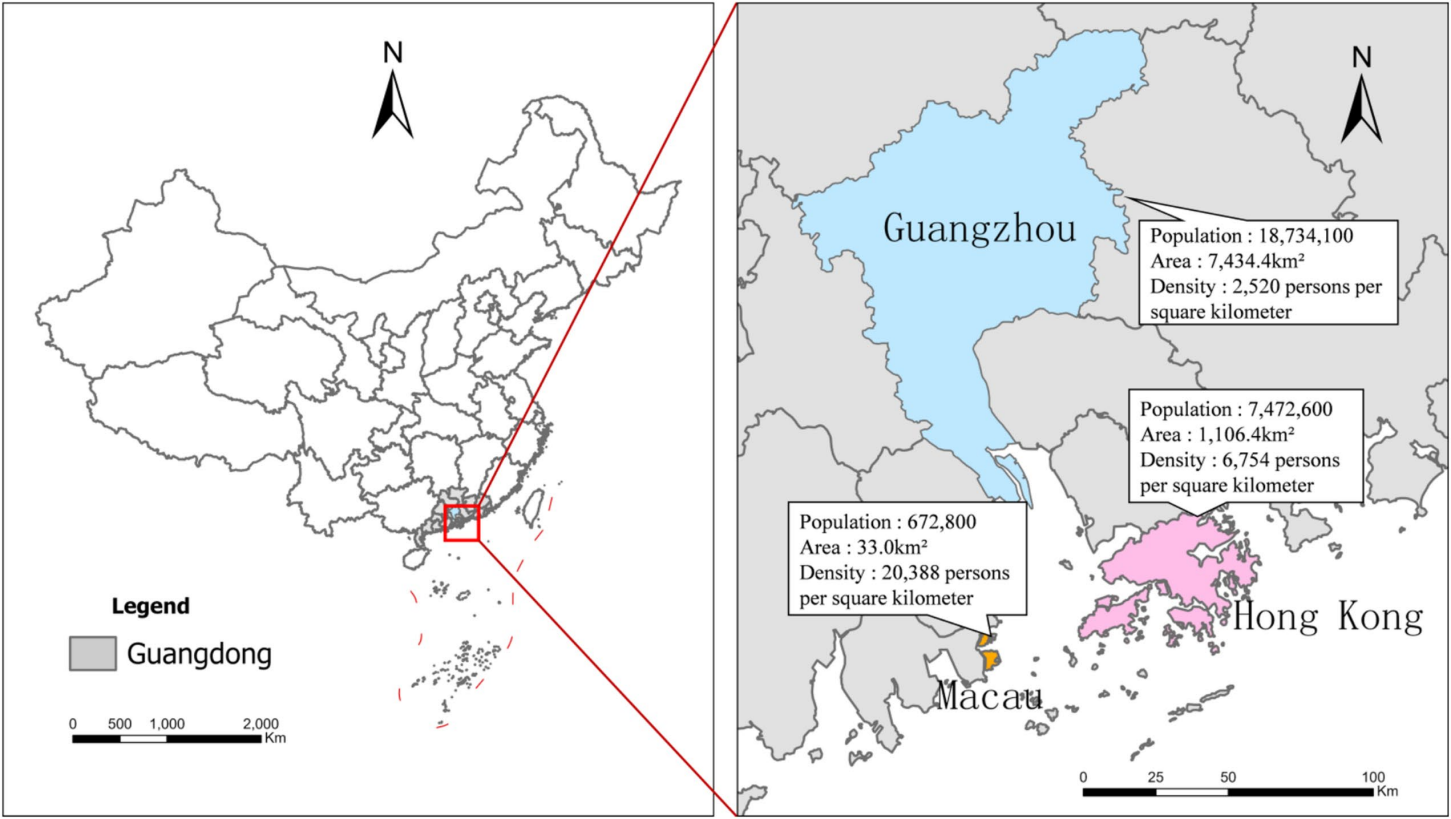
Geographic context and population density of Guangdong, Guangzhou, Hong Kong, and Macau in 2022.

The Greater Bay Area of China, encompassing Guangdong Province, Hong Kong SAR, and Macau SAR, presents a quasi-natural experiment setting for studying the impact of different political systems on COVID-19 response effectiveness. They are situated close to one another, with distances not exceeding approximately 100 km. Figure 1 illustrates the geographic context of Guangdong, Guangzhou, Hong Kong and Macau, which also highlights the striking gradient in population density across the region in 2022. This region offers a unique research opportunity as it contains three distinct administrative entities that share deep cultural and historical ties, yet operate under different political frameworks (29). In this study, a comprehensive comparative analysis is conducted across the three regions. When analyzing prevention policies and social media discourse, Guangdong’s figures are treated as a whole to provide a more complete and integrated perspective, given the consistency of policies across the province. However, for city-level comparisons—such as infection rates, population density, GDP, and hospital bed availability—Guangzhou, as the provincial capital, is used to represent Guangdong in direct comparison with Hong Kong and Macau. This configuration allows us to control for geographical and cultural variables while focusing on how different governance approaches influenced pandemic outcomes. The three regions represent diverse political structures. Guangdong exemplifies a society-mobilizing mainland system. Hong Kong, as a former British colony, demonstrates a more market-facilitated approach. The small gambling city of Macau, which was once occupied by Portugal, operates under an elite-driven targeted-control governance model. Recent research highlights how these jurisdictional differences led to varying response strategies despite their geographical proximity and shared cultural heritage (30).

### 1.2 Data collection, preprocessing, and cleaning

**Figure 2:**
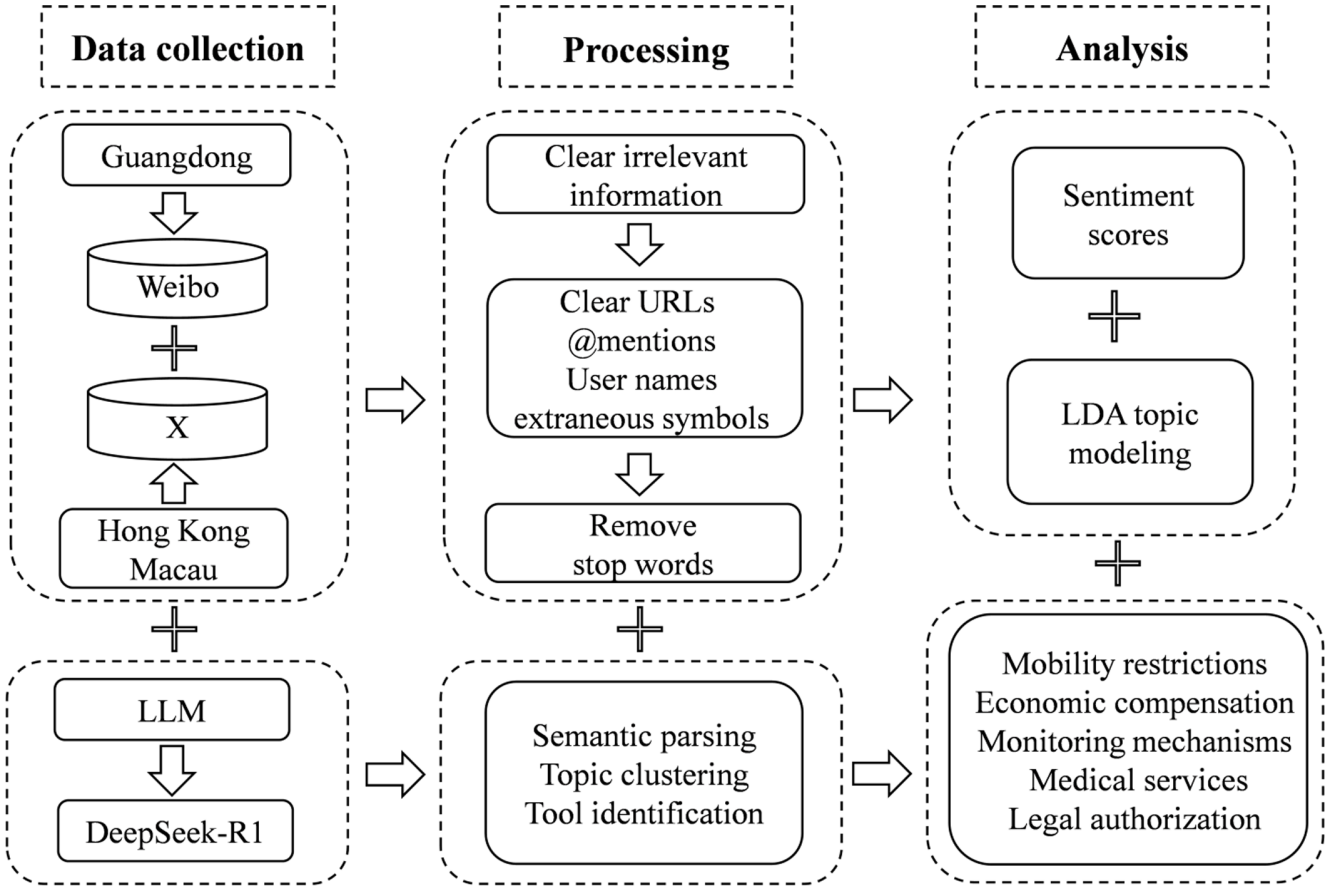
Flowchart of data collection, preprocessing, and analysis procedures.

Our study employed an innovative mixed-method approach to data collection, combining advanced comprehensive policy analysis, statistical analysis and social media analysis (Figure 2). The policy analysis component of our research drew from two primary sources: official government documents and existing academic literature. We systematically reviewed policy announcements, press releases, and regulatory guidelines published by relevant authorities in each region, supplemented by peer-reviewed studies analyzing these policies. For epidemiological data collection, we developed specialized Python scripts to automatically extract daily infection statistics from the official websites of the Guangzhou City Government and the National Health Commission (for Hong Kong and Macau), noting that although some discrepancies exist, this is currently the most accurate data available. To ensure high data consistency and accuracy, automated cross-validation protocols were implemented. Beyond analyzing and categorizing the distinct COVID-19 containment policy approaches implemented across Guangzhou, Hong Kong, and Macau, our research methodology employed Python-based web crawlers to systematically collect and compile daily infection statistics and 75,870 social media posts throughout a 3-year period from December 8, 2019, to December 7, 2022. The complete data-collection phase lasted 2 months, spanning iterative pilot runs, rate-limit negotiations with platform APIs, and systematic full-scale harvesting to guarantee temporal completeness. This method enables a comprehensive examination of both epidemiological trends and public discourse across these three interconnected Greater Bay Area cities. While acknowledging the inherent limitations of cross-platform comparison, our choice to analyze Weibo data for Guangzhou and X data for Hong Kong and Macau reflects the practical reality of social media usage patterns in these regions. Given that mainland Chinese citizens have limited access to X due to network restrictions, while Hong Kong and Macau residents predominantly use international social media platforms rather than mainland-based ones, this cross-platform approach represents the most feasible method for capturing authentic public discourse in each region. Although this methodological compromise introduces certain analytical challenges, it provides the most representative sample of genuine public sentiment within each region’s distinct information ecosystem.

This study period encompasses the complete trajectory of China’s COVID-19 pandemic response, spanning from the initial outbreak in late 2019 through the pivotal policy shift marked by the State Council’s “Notice on Further Optimizing the Implementation of COVID-19 Prevention and Control Measures” (commonly known as the “New Ten Points”) in December 2022, which fundamentally altered the nation’s approach to pandemic management and marked the beginning of China’s transition away from its strict “zero-COVID” policy. This dataset comprised 20,591 Weibo posts from Guangdong and 55,279 X posts (53,919 from Hong Kong and 1,360 from Macau). Following established methodologies, we obtained data through officially authorized channels to ensure data authenticity while adhering to platform guidelines (31). Specifically, we applied for and received developer account access from Weibo and X, and collected data via their official API interfaces, ensuring full compliance with their data use policies for non-commercial third-party research. We rigorously adhered to the API’s rate limits and data scope restrictions (such as not accessing non-public posts). All user-identifiable information and profile data were removed from the datasets during processing. Strict data management protocols were also implemented to ensure no risk of privacy infringement. The data processing procedure consisted of four steps. First, we removed promotional content and irrelevant texts using automated filtering algorithms. Second, we cleaned the data by eliminating URLs, @mentions, usernames, and extraneous symbols. Third, we implemented text segmentation for Chinese language processing, a crucial step for COVID-19 sentiment analysis (32). Finally, we removed stop words to prepare the text for analysis. This entire data-cleaning pipeline was executed over a concentrated two-week period, during which iterative quality checks were performed to ensure that the final corpus was free of duplicates, spam, and non-COVID-19 content. Keywords used for data collection included region-specific terms combined with “COVID-19” and “epidemic” in both Chinese and English to ensure comprehensive coverage.

### 1.3 Data analysis

Following the “policy style-effect-feedback” framework, the empirical analysis employed a mixed-methods approach to examine the effectiveness of different COVID-19 policy styles across the three Greater Bay Area cities. The study period spanned from January 2020 to December 2022, encompassing multiple pandemic waves. We first conducted descriptive statistical analysis to understand regional variations in COVID-19 cases, examining both cumulative confirmed cases and daily new infections. This analysis revealed significant scale differences between regions, with Hong Kong’s cumulative cases far exceeding Guangzhou and Macau. To capture temporal dynamics, we performed monthly trend analysis and phase-specific assessments, dividing the pandemic into three distinct periods: Initial Outbreak, Containment Phase, and Omicron Phase. For quantitative analysis, we employed an interrupted time series model using segmented regression to assess how epidemic trends shifted across different phases. This comprehensive analytical approach, supplemented by visualization techniques including log-scale analysis of cumulative COVID-19 cases, enabled us to establish clear linkages between governance approaches and pandemic outcomes while effectively controlling for regional variations and temporal factors.

We also combined sentiment analysis with topic modeling to provide a nuanced understanding of public response to COVID-19 across the three regions. For sentiment analysis, we utilized the Baidu Sentiment Analysis API, which has been specifically trained on Chinese language content. Previous research has demonstrated this approach’s particular effectiveness in analyzing Chinese social media content during public health crises (33). The sentiment scores were categorized into three levels: positive (>0.6), neutral (0.4–0.6), and negative (<0.4). For topic modeling, we employed the Latent Dirichlet Allocation (LDA) algorithm, which has been proven effective in uncovering latent themes in COVID-19-related social media discussions (34). The LDA model was optimized through iterative testing to identify the optimal number of topics that would provide meaningful insights while avoiding redundancy. We supplemented this with temporal analysis to track sentiment and topic evolution from December 2019 to December 2022. To ensure robust results, we implemented several validation measures. First, we conducted cross-validation of our sentiment analysis results using a subset of manually coded posts. Second, we employed multiple human coders to verify the coherence and interpretability of the LDA-generated topics. Following established protocols, we also calculated inter-rater reliability scores to ensure consistency in our topic interpretations (35). Alongside public response analysis, we adopted a natural language processing (NLP) approach based on large language models (LLMs) to analyze official policy texts. The DeepSeek-R1 large-scale language model excels in semantic understanding, contextual reasoning, and knowledge association. We developed a classification system covering five categories of policy tools: mobility restrictions, economic compensation, monitoring mechanisms, medical services, and legal authorization. Using semantic parsing, topic clustering, and tool identification, we aggregated policy tool deployment frequency by jurisdiction, revealing significant regional differences in how these tools were applied. The comparative analysis framework was structured to examine three key aspects across regions: sentiment distribution, topic patterns, and how they respond to policy implementation. For sentiment analysis, we calculated average sentiment scores and the percentage distribution of positive, negative, and neutral reactions. Using LDA topic modeling, we identified and quantified region-specific topics and their respective proportions. This analytical approach enabled us to systematically compare public responses across Guangdong, Hong Kong, and Macau while accounting for their distinct contextual characteristics. This approach revealed how different governance styles shaped public response: society-mobilizing tools facilitated stable implementation but potentially limited individual expression, market-facilitated tools encouraged active debate but risked social tension, and targeted-control tools maintained economic focus but showed vulnerability to public sentiment shifts (36).

## 2 Results

### 2.1 Policy style: key features, primary tools, and societal response

The COVID-19 responses in Guangdong, Hong Kong, and Macau demonstrate distinct policy styles shaped by their institutional infrastructures and governance traditions. We argue that these approaches can be characterized as society-mobilizing, market-facilitated, and targeted-control, respectively, as shown in Table 1. To enrich the above description with systematic policy-text evidence, we also extracted and classified COVID-19 policy documents issued by Guangzhou (*n* = 150 sentences), Hong Kong (*n* = 40), and Macau (*n* = 33) between January 2020 and December 2022. Using an LLM-based topic-modeling pipeline powered by the DeepSeek API, we coded each sentence for the presence of eight mutually exclusive policy-tool categories: mobility restrictions, economic compensation, surveillance mechanisms, medical services, legal authorization, supply-chain support, risk communication and “other.” Frequencies were normalized by the total number of sentences per jurisdiction and are reported in Table 2.

**Table 1:** Comparison of COVID-19 policy styles in three regions.

Region	Policy style	Key features	Primary tools	Societal response
Guangdong	Society-mobilizing	Centralized coordination; mass mobilization; sacrifice for collective good	Community grid management; digital surveillance; universal testing	High compliance through social pressure; strong state capacity
Hong Kong	Market-facilitated	Civil society engagement; public pressure responsiveness; fragmented implementation	Voluntary measures; public-private partnerships; targeted restrictions	Active civil society participation; mixed public trust
Macau	Targeted-control	Result-oriented; industry-integrated; flexible adaptation	Casino-integrated measures; universal testing; border controls	High compliance through economic incentives; pragmatic acceptance

**Table 2:** Deployment of policy tools across regions.

Policy tools	Guangdong	Hong Kong	Macau	Total
Mobility restrictions	8 (5.3%)	2 (5.0%)	14 (42.4%)	24 (10.8%)
Economic compensation	19 (12.7%)	1 (2.5%)	2 (6.1%)	22 (9.9%)
Surveillance mechanisms	52 (34.7%)	13 (32.5%)	4 (12.1%)	69 (30.9%)
Medical services	13 (17.4%)	15 (37.5%)	2 (6.1%)	30 (13.4%)
Legal authorization	5 (3.3%)	9 (22.5%)	11 (33.3%)	25 (11.2%)

Guangdong adopted a society-mobilizing approach, characterized by centralized coordination and mass mobilization, where societal interests consistently superseded individual rights (37). The key features of this approach included extensive centralized coordination and the willingness to implement sacrificial measures for collective benefit. This manifested through primary tools such as the community grid management system and comprehensive digital surveillance mechanisms. The deployment of neighborhood committees for contact tracing and the implementation of health QR codes achieved remarkably high compliance through social pressure and deeply rooted cultural values of collectivism (38). The government’s strong state capacity enabled the coordination of mass testing campaigns, where cities with millions of residents were tested within days, demonstrating the effectiveness of collective action in crisis management (37). In terms of topic modeling results, “surveillance mechanisms” dominated, accounting for 34.7% (52/150) of all policy sentences, followed by “supply-chain support” (17.4%, 26/150) and “mobility restrictions” (12.0%, 18/150). This distribution corroborates the society-mobilizing narrative: the state prioritized digital tracking, grid management and rapid lockdown logistics over explicit legal mandates or economic subsidies. By contrast, only 5.3% (8/150) of Guangdong’s sentences referenced “economic compensation,” indicating that financial relief was rhetorically peripheral.

Hong Kong’s response exemplified a market-facilitated style, marked by significant civil society engagement and responsiveness to public pressure. This approach led to a more fragmented implementation process, heavily influenced by public opinion and civil society dynamics (39). Notably, Hong Kong faced pressure from society and the business sector, which prevented the implementation of universal nucleic acid testing, while both Guangdong and Macau conducted multiple rounds of universal testing (40, 41). The region’s primary tools centered on voluntary measures, public-private partnerships, and targeted restrictions rather than universal mandates. This was evident when the closure of certain border checkpoints was implemented only after healthcare workers initiated strike action (41). The voluntary nature of mask-wearing, which achieved widespread adoption through community initiatives rather than government mandates, further illustrated this approach. The government operated in a low-trust environment that required continuous negotiation with various stakeholders, resulting in active civil society participation but mixed levels of public trust in official measures (42). Our NLP-based coding reveals that Hong Kong exhibited the highest relative share of “legal authorization” sentences (22.5%, 9/40), reflecting the administration’s need to anchor measures in explicit statutory powers amid intense judicial and societal scrutiny. “Medical services” constituted 37.5% (15/40) of sentences, underscoring the territory’s emphasis on hospital capacity and targeted care instead of blanket restrictions. Conversely, “surveillance mechanisms” (32.5%, 13/40) remained substantial but were framed within privacy safeguards, while “mobility restrictions” appeared sparingly (5.0%, 2/40), consistent with the market-facilitated reluctance to impose universal lockdowns.

Macau’s targeted-control approach distinguished itself through a unique combination of strong government control and practical flexibility. This result-oriented style integrated industry considerations, particularly drawing from its experience in casino management (43). The region’s primary tools included casino-integrated measures, universal testing protocols, and strict border controls. Authorities implemented targeted restrictions while maintaining essential economic activities, demonstrating practical adaptation to changing circumstances. The government’s coordination with casino operators led to sophisticated contact tracing and testing systems (44), complemented by economic support for affected sectors (45). This pragmatic style facilitated rapid policy adjustments based on empirical outcomes rather than ideological considerations, resulting in high compliance through economic incentives and pragmatic acceptance among the population. Content analysis of Macau’s policy corpus shows the largest proportion of sentences devoted to “legal authorization” (33.3%, 11/33), signaling the government’s strategic use of precise legal instruments to legitimize rapid, industry-specific interventions. “Mobility restrictions” constituted 42.4% (14/33) of sentences, almost all linked to border and casino-entry controls, while “surveillance mechanisms” appeared less frequently (12.1%, 4/33) than in Guangzhou or Hong Kong. Only 6.1% (2/33) of sentences discussed “economic compensation,” indicating that financial support was largely implicit within the casino-concession framework rather than explicit in policy discourse.

These distinct policy styles significantly influenced the regions’ pandemic outcomes. While all three regions achieved relative success in controlling COVID-19 compared to many Western countries, Hong Kong reported substantially higher cumulative case numbers than Guangzhou and Macau. The variations in their policy styles and effectiveness reflect deeper institutional characteristics and governance traditions, offering valuable insights for understanding crisis response in different political contexts (46). These styles do not operate in a vacuum; they are enabled, or constrained, by the underlying institutional capacity that determines which tools can be swiftly and credibly deployed.

### 2.2 Policy effectiveness: analysis of infection patterns

The analysis of COVID-19 infection patterns across Guangzhou, Hong Kong, and Macau reveals how institutional capacity embedded in each policy style translated tools and public response into measurable epidemiological outcomes. Through a rigorous examination of 1,050 days of pandemic data, statistical analysis definitively exposes the profound differences in COVID-19 transmission patterns across the three regions. These divergent approaches—society-mobilizing, market-facilitated, and targeted-control, respectively—produced dramatically different infection trajectories. As shown in Table 3, Hong Kong recorded the highest infection levels, with daily new cases averaging 442.95 and reaching peaks of up to 31,368 cases, ultimately accumulating to a maximum of 465,099 total cases. Guangzhou demonstrated moderate containment success, maintaining a lower average of 31.03 daily new cases, though still experiencing significant spikes of up to 1,650 new cases in peak periods, with cumulative cases reaching 32,583. In stark contrast, Macau’s stringent border control measures proved highly effective, resulting in remarkably low transmission rates with an average of merely 0.79 new cases daily, never exceeding 61 cases in a single month, and maintaining a maximum cumulative case count of 826 throughout the studied period. To complement the infection data, Table 4 offers a detailed comparison across the three regions, including infection counts, infection rates, and key contextual variables such as population density, annual passenger throughput, hospital bed availability, and GDP loss. It is shown that Macau has an exceptionally high population density of 20,388 persons per km^2^ but recorded only 826 infections, resulting in the lowest infection rate of 122.77 per 100,000 people, while also suffering the most severe GDP loss (47–53). Guangzhou, despite having the largest annual passenger throughput at over 26 million and a moderate population density of 2,520 persons per km^2^, reported 32,583 infections with an infection rate of 173.91. Meanwhile, Hong Kong, with a population density of 6,754 persons per km^2^ and significantly fewer passengers at approximately 5.6 million annually, experienced the highest infection count of 465,099 and an infection rate exceeding 6,200 per 100,000 people. These contrasts reveal that neither high population density nor large mobility volumes necessarily translate into higher infection numbers. In fact, factors traditionally considered to increase infection risk did not align with the observed infection metrics, highlighting that effective policy measures, rather than demographic or mobility factors alone, are likely the decisive elements in managing the spread of COVID-19.

**Table 3:** Descriptive statistics by region.

Region	Variable*	Mean	Median	SD	Min	Max
Guangzhou	cumulative	2006.19	1410.50	3078.35	0	32,583
	new_cases	31.03	2.50	163.80	0	1,650
Hong Kong	cumulative	101772.70	11920.50	158464.20	0	465,099
	new_cases	442.95	14	2090.04	0	31,368
Macau	cumulative	163.67	54	257.63	0	826
	new_cases	0.79	0	4.67	0	61

*Cumulative refers to monthly cumulative confirmed cases; new_cases refers to monthly newly confirmed cases.

**Table 4:** Regional contextual factors and COVID-19 infection metrics by the end of 2022.

Region	Population density in 2022 (persons/km^2^)	Passenger throughput in 2022	Hospital beds (per 1,000 people)	Number of infections by 2022	Infection rate (per 100,000 people)	GDP Loss (compared to 2019)
Guangzhou	2,520	26,104,989	5.4	32,583	173.91	+22.05%
Hong Kong	6,754	5,656,000	4.9	465,099	6224.05	−00.94%
Macau	20,388	599,185	2.8	826	122.77	−54.64%

**Figure 3:**
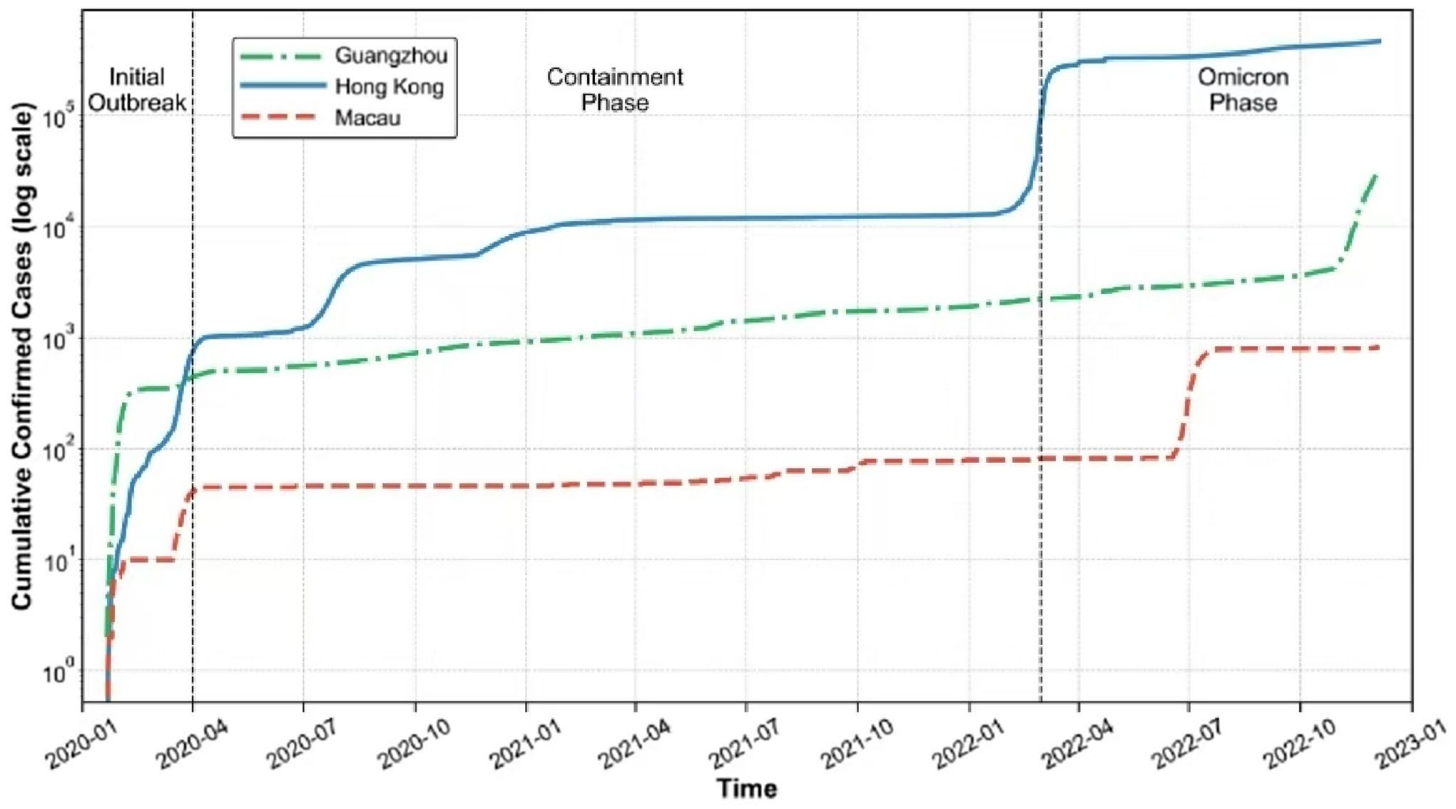
Cumulative confirmed COVID-19 cases (log scale) in Greater Bay Area Cities with phase divisions.

We also utilize the quantitative analysis tool, interrupted time series analysis, to provide a time series perspective that illustrates the effects of policies and epidemic transmission trends across different regions and stages of the pandemic, assessing the effectiveness of pandemic policies in Guangzhou, Hong Kong, and Macau (Table 5 and Figure 3). The analysis delineates three key periods in the pandemic timeline, consistent with established periodization in prior studies: Initial Outbreak (January to March 2020), the Containment Phase (April 2020 to February 2022), and the Omicron Phase (March to December 2022) (54–56). Each phase exhibits distinct epidemiological trends and policy responses, as evidenced by segmented regression results and cumulative case data. This approach enhances our understanding of how varying policy styles impacted the pandemic response in these regions.

**Table 5:** Segmented regression results from interrupted time series analysis.

Region	Trend (Phase 1)	Trend (Phase 2)	Trend (Phase 3)
Guangzhou	−0.164	0.004	1.9783***
Hong Kong	0.561	0.773**	−13.09***
Macau	0.018	0.0001	−0.0027

***p < 0.01, **p < 0.05.

According to Table 5 and Figure 3, during the Initial Outbreak phase, the ITS analysis shows that Guangzhou’s COVID-19 transmission trend exhibited a slight decline (Trend = −0.164), indicating that the early containment measures had already started to take effect and demonstrating the rapid response capability of its society-mobilizing governance model. In contrast, Hong Kong experienced an upward transmission trend (Trend = 0.561), likely due to a more fragmented approach to pandemic governance that resulted in less stringent policy implementation and less effective suppression of viral spread. Macau, meanwhile, maintained a remarkably stable trend (Trend = 0.018), which reflects the strong policy enforcement and early effectiveness of its elite-driven governance model in curbing the spread. While these results are not statistically significant, they still provide insight into the early development trajectories in each region. The log-scale visualization of cumulative COVID-19 cases in Figure 3 continues to show a rapid escalation in case numbers, especially in Guangzhou, with Hong Kong mirroring this trend and Macau’s case count increasing more slowly, fitting the differences highlighted by ITS analysis. This phase underscores the influence of governance modes and the urgency with which public health measures need to be implemented.

As the pandemic progressed into the Containment Phase, Guangzhou’s transmission trend stabilized (Trend = 0.004), suggesting that the “precision prevention and control” strategy under its society-mobilizing model effectively contained further spread. Hong Kong’s increasing trend (Trend = 0.773, *p* < 0.05) points to ongoing challenges, such as fragmented policy execution and lower public compliance, which hindered effective control over the outbreak. Macau’s trend remained almost unchanged (Trend = 0.0001), signaling that its elite-driven governance and strong policy enforcement continued to suppress transmission. Again, even the non-significant results provide a valuable reflection of the direction and effectiveness of the interventions taken. In all three regions, relatively low and steady case numbers in this phase reflect the impact of timely and effective interventions, such as lockdowns, social distancing, and mask mandates. This period illustrates how coordinated public health responses—tailored to distinct local governance modes—can lead to stabilization and reduced spread.

During the Omicron Phase from March to December 2022, the ITS analysis reveals a sharp rise in Guangzhou’s transmission trend (Trend = 1.9783, *p* < 0.01), likely due to the highly infectious nature of the Omicron variant, though policy responses still managed to exert a strong suppressive effect. Hong Kong, in contrast, saw a significant decline in the transmission trend (Trend = −13.09, *p* < 0.01), indicating that more stringent containment measures were implemented, albeit with continued volatility in case numbers that reflects the challenges of managing high-transmission variants within its governance context. Macau’s trend showed a slight decrease (Trend = −0.0027), demonstrating the continued effectiveness and adaptability of its elite-driven model in containing new waves. The significant results in this phase highlight the dramatic policy impacts, while the non-significant result for Macau still suggests persistent control over transmission. The log-scale visualization of cumulative cases still shows a steep increase for Hong Kong and Guangzhou, while Macau’s trajectory remains close to the horizontal axis, echoing the ITS findings and underscoring the value of strict border controls and rapid interventions.

The divergent trends observed in these three cities exemplify the varying public health strategies employed and their subsequent outcomes. Guangzhou’s society-mobilizing approach emphasized community responsibility and a coordinated response to public health challenges, allowing for effective management of imported Omicron variants. Conversely, Hong Kong’s market-facilitated strategy, which initially prioritized individual freedoms and less stringent measures, led to a late surge in cases, illustrating the difficulties encountered when community transmission escalates. In contrast, Macau’s targeted-control approach, characterized by strict border controls and rapid response measures, proved highly effective in minimizing transmission throughout the pandemic, resulting in the lowest case numbers among the three regions. Despite their geographical proximity and socioeconomic similarities, the stark differences in cumulative infection numbers underscore the profound impact of distinct policy approaches. This comparative analysis validates the selection of the Greater Bay Area as a quasi-natural experiment setting, where similar underlying conditions amplify the observable effects of divergent policy choices on pandemic outcomes.

### 2.3 Policy feedback: public opinion analysis of COVID-19 control measures

The analysis of public sentiment toward COVID-19 policies across Guangdong, Hong Kong, and Macau exposes nuanced patterns deeply rooted in their respective political-social structures, demonstrating how different governance approaches shaped public response to pandemic management. In Guangdong, where society-mobilizing tools prevail, sentiment scores remained consistently highest (0.54) and most stable, with 51.64% positive reactions and 41.93% negative responses (Table 6). This stability reflects strong social cohesion and widespread acceptance of control measures, as citizens prioritized collective welfare over individual interests. The LDA model revealed focused attention on systematic pandemic control, with key topics including overseas case imports (Topic 1: 32.5%) and infection monitoring (Table 7). Text analysis showed frequent mentions of “nucleic acid testing,” “prevention,” and “work resumption,” indicating a comprehensive approach combining health measures with economic recovery. Even during periods of strict control, Guangdong’s sentiment remained stable, demonstrating how socially mobilized values facilitated consistent policy implementation and public compliance.

**Table 6:** Proportion of emotional text by region.

Region	Positive emotion	Neutral emotion	Negative emotion	Mean
Guangdong	51.64%	6.43%	41.93%	0.54
Hong Kong	44.62%	4.36%	51.02%	0.46
Macau	44.56%	5.22%	50.22%	0.47

**Table 7:** Topic-word probability distribution and topic classification of LDA models in the Greater Bay Area.

Region	Topic	Topic-word distribution	Topic type
Guangdong	1	0.325”cases” + 0.033”confirmed” + 0.025”new” + 0.021”imported” + 0.020”cumulative” + 0.020”overseas” + 0.015*"discharged”	Imported cases
	2	0.056”cases” + 0.031”confirmed” + 0.028”pneumonia” + 0.016”epidemic” + 0.016”COVID” + 0.014”infection” + 0.014*"newly reported”	Infection cases
	3	0.014”epidemic” + 0.009”community” + 0.009”Guangdong Province” + 0.007”return to work” + 0.006”locked down” + 0.005”number” + 0.005*"passengers”	Epidemic prevention and control
	4	0.026”prevention and control” + 0.021”epidemic” + 0.019 “personnel” + 0.014”work” + 0.009″ testing” + 0.008”ensure”+ 0.008*"pneumonia”	Isolation and testing
	5	0.018”epidemic” + 0.008”enterprises” + 0.006”work” + 0.005”Guangdong Province” + 0.005”work resumption” + 0.004”Wuhan”+ 0.004*"China”	Enterprise work resumption
Hong Kong	1	0.055”Hong Kong” + 0.049”epidemic” + 0.009”vaccine” + 0.008”government” + 0.008”anti-epidemic” + 0.007”citizens”+ 0.006*"COVID-19”	Vaccine administration
	2	0.040”Hong Kong” + 0.040”cases” + 0.038”epidemic” + 0.028”confirmed” + 0.014”newly added” + 0.013 “pneumonia”+ 0.013*"death”	Local epidemic
	3	0.035”epidemic” + 0.031”Hong Kong” + 0.009 “pneumonia” + 0.007”COVID-19″ + 0.006”quarantine” + 0.005”Wuhan”+ 0.005*"hospital”	Epidemic prevention and control
	4	0.040”Hong Kong” + 0.033”epidemic” + 0.032”China” + 0.008”Shanghai” + 0.007”United States” + 0.007 “country” + 0.007*"Taiwan”	Mainland epidemic
	5	0.046”Hong Kong” + 0.041”epidemic” + 0.014”not” + 0.014”United States” + 0.010”Taiwan” + 0.007”China” + 0.006*"mainland”	Anti-epidemic measures
Macau	1	0.026”Macau” + 0.025”epidemic” + 0.011”China” + 0.009 “Hong Kong” + 0.006”COVID-19″ + 0.005”Taiwan” + 0.005*"cases”	Epidemic situation
	2	0.048”Macau” + 0.032”epidemic” + 0.010”casino” + 0.010”Hong Kong” + 0.005”pneumonia” + 0.005”China” + 0.005*"latest news”	Impact on casinos
	3	0.036”Macau” + 0.030”epidemic” + 0.021”cases” + 0.011”Hong Kong” + 0.009”mainland” + 0.008”China” + 0.008*"nucleic acid”	Epidemic prevention measures
	4	0.053”Macau” + 0.037”epidemic” + 0.028”cases” + 0.012”confirmed” + 0.011”Hong Kong” + 0.009”China” + 0.006*"mainland”	Mainland epidemic
	5	0.074”cases” + 0.032”epidemic” + 0.030”Macau” + 0.015”Hong Kong” + 0.013”cumulative” + 0.013 “confirmed” + 0.012*"China”	Local epidemic

Hong Kong’s market-facilitated environment produced markedly different results, with the lowest average sentiment (0.46) and highest volatility among the three regions (Table 6). This city showed the highest proportion of negative sentiments (51.02%), reflecting intense public scrutiny of government decisions. Topic modeling revealed a distinct focus on policy criticism, vaccine hesitancy, and international developments, with key terms like “government,” “United States,” “Taiwan,” and “citizens” frequently appearing in social media discussions. The LDA analysis demonstrated Hong Kong’s unique concern pattern, with significant attention to vaccine-related issues (Topic 1: 5.5%) and local case monitoring (Topic 2: 4.0%) (Table 7). This reflects Hong Kong’s position as an international city where public discourse actively engages with both local and global perspectives. The market-facilitated characteristics led to rapid sentiment shifts in response to policy adjustments, particularly regarding vaccine deployment and border control measures.

Macau’s approach, characterized by targeted-control tools, yielded moderate sentiment scores (0.47) with a distinct temporal pattern of initial stability followed by increasing volatility (Table 6). The region’s discourse was dominated by economic concerns, with the LDA model showing significant attention to casino impacts and tourism effects (Topic 2: 4.8%) (Table 7). Text analysis revealed frequent discussion of economic recovery initiatives, with featured keywords like “casino,” “tourism,” “China Mainland,” “Hong Kong,” and “economy” appearing prominently in social media posts. Macau’s public sentiment reflected its unique position as a gaming and tourism hub, with public discourse focusing heavily on economic impacts rather than health measures. The territory’s elite-driven decision-making process initially maintained stability, but sentiment scores fluctuated significantly when economic recovery measures failed to meet public expectations, demonstrating the vulnerability of targeted-control tools to economic performance metrics.

**Figure 4:**
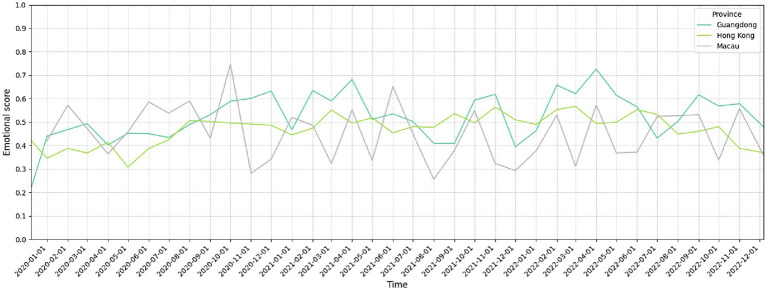
Monthly average sentiment scores in Guangdong, Hong Kong, and Macau.

Due to the relatively low number of monthly new infections compared to the population base in all three regions, their respective sentiment scores did not fluctuate with the number of new cases (Figure 4). However, the trends and topic distributions still formed their own characteristics. The comparative analysis suggests that while society-mobilizing approaches may facilitate stable policy implementation, they potentially limit individual expression; market-facilitated environments encourage active policy debate but risk social tension; and targeted-control tools maintain economic focus but remain vulnerable to public sentiment shifts when economic goals are unmet. The temporal analysis from December 2019 to December 2022 further revealed how these structural differences influenced public response to various pandemic phases, with Guangdong maintaining the most consistent sentiment trajectory despite policy changes. The results demonstrate that social media sentiment analysis can effectively capture the nuanced ways in which different political-social structures shape public response to crisis management policies.

## 3 Discussion

Mechanistically, our findings show how institutional capacity, tool choice and public response operate inside the “policy style-effect-feedback” loop. Guangdong’s centralized capacity enabled its society-mobilizing style to deploy city-wide nucleic-acid testing within 48 h, while neighborhood committees leveraged state legitimacy to yield 51.6% favorable sentiment and keep the daily mean at 31 cases. Hong Kong’s fragmented capacity confined its market-facilitated style to voluntary measures and public-private partnerships, generating 51.0% negative sentiment and an average of 443 daily cases. Macau’s elite-driven capacity translated its targeted-control style into casino-integrated contact tracing and rapid border closures, producing moderate sentiment (0.47) and the lowest caseload (0.79). Each outcome fed back to reinforce or erode the institutional capital available for the next wave, illustrating how the same causal chain produces divergent epidemiological results when authority configuration and societal trust vary.

To move beyond description, we synthesize legitimacy, effectiveness and crisis management into a single theoretical pathway that links political structure to implementation efficacy through three sequential filters. First, authority configuration—centralized in Guangdong, negotiated among societal actors in Hong Kong, or elite-directed in Macau—determines which policy tools are legally and logistically deployable (42). Second, societal trust modulates the speed and scale of voluntary compliance; where trust is high, as in Guangdong, even intrusive measures such as digital health codes are accepted, whereas in Hong Kong prior legitimacy deficits amplified resistance to universal testing mandates (12). Third, policy feedback either replenishes or drains the institutional resources required for subsequent waves. Empirically, Guangdong’s neighborhood-committee network converted central directives into granular compliance; positive sentiment and successful containment channeled public gratitude back into state capacity. Conversely, Hong Kong’s tripartite bargaining among government, medical unions and business associations slowed decision-making, while negative sentiment after the 2019 protests eroded compliance during the Omicron surge (46). In Macau, the casino-state nexus allowed swift border closures, yet when promised tourism subsidies lagged, sentiment volatility undermined trust and threatened future compliance (43).

Our Greater Bay Area quasi-experiment therefore corroborates two recent comparative findings while adding temporal granularity unavailable in cross-national aggregates. Legitimacy crises in low-trust polities hinder containment, but the same polity can experience both crisis and correction depending on how authority configuration and feedback interact across pandemic phases (42). Value conflicts between freedom and security drive policy variation in democracies, and this tension is resolved differently within a single metropolitan region under centralized mobilization versus pluralist negotiation (10). Methodologically, combining epidemiological interrupted time-series with sentiment-based feedback loops offers a template for analyzing real-time policy recalibration that single-country case studies rarely provide (21).

## 4 Conclusion

This study demonstrates that the effectiveness of pandemic governance depends on the dynamic interplay within the “policy style-effect-feedback” framework. Our comparative analysis of COVID-19 responses across China’s Greater Bay Area reveals how different political systems within the same cultural context can produce distinct pandemic management outcomes. This study shows that while all three approaches—society-mobilizing, market-facilitated, and targeted-control—achieved relative success by global standards, their effectiveness varied significantly based on institutional infrastructure and governance traditions. The findings suggest that successful pandemic responses depend not just on policy tools but also on the alignment between governance approaches and existing social structures. Guangdong’s society-mobilizing approach demonstrated how deeply embedded institutional infrastructure can facilitate consistent policy implementation, while Hong Kong’s market-facilitated model highlighted the challenges of crisis management in low-trust environments. Macau’s targeted-control approach offered insights into balancing strict control measures with economic considerations.

This comparison is particularly valuable for informing early-stage pandemic response strategies when medical interventions are limited and viral characteristics remain unclear. Our results show that, during such critical periods, the choice of policy style can significantly impact the trajectory of transmission, as evidenced by the magnitude and statistical significance of trends observed in different phases and regions. These insights provide crucial guidance for policymakers facing similar circumstances of uncertainty and urgency. The study demonstrates that different governance approaches can be effective when properly aligned with local institutional capabilities and social contexts, offering a framework for rapid decision-making in future disease outbreaks.

As countries worldwide transition to post-pandemic management, new research directions emerge. Further studies are needed to examine how these different policy styles adapt to the post-containment phase, particularly investigating their effectiveness in managing public health while supporting economic recovery and social equity. Of particular interest would be comparative analyses of how different governance systems balance endemic COVID-19 management with societal reopening, and how initial policy choices influence long-term public health outcomes and social resilience. These insights will be crucial for developing comprehensive frameworks that can guide policy responses across the full spectrum of pandemic management, from the initial outbreak to the endemic phase. While our findings are grounded in this specific regional context, we recognize that the external applicability to other regions requires careful consideration of local institutional and cultural differences. This study is limited by its focus on three cases within a shared cultural and geographic environment, which may constrain the generalizability of the conclusions. Differences in political structures, social norms, and public health infrastructure elsewhere could influence how these governance mechanisms operate. Nonetheless, the core framework offers broadly relevant insights into pandemic governance that can inform analysis and policy design in diverse settings. Therefore, while caution is warranted in direct extrapolation, we believe the theoretical pathway developed here holds substantial potential for application beyond the Greater Bay Area. This integrated framework offers a robust theoretical pathway for analyzing crisis management beyond COVID-19.

## Data Availability

The raw data supporting the conclusions of this article will be made available by the authors, without undue reservation.
